# A222 CLINICAL EFFECTIVENESS AND SAFETY OF USTEKINUMAB IN YOUTH WITH REFRACTORY INFLAMMATORY BOWEL DISEASE IN SAUDI ARABIA: A RETROSPECTIVE COHORT STUDY

**DOI:** 10.1093/jcag/gwad061.222

**Published:** 2024-02-14

**Authors:** A A Alhadab, A Almarhoon, A AlAlwan, A Hammo

**Affiliations:** Pediatric Gastroenterology, McMaster University Faculty of Health Sciences, Hamilton, ON, Canada; King Fahad Specialist Hospital Dammam, Dammam, Eastern, Saudi Arabia; King Fahad Specialist Hospital Dammam, Dammam, Eastern, Saudi Arabia; University of Arkansas for Medical Sciences, Little Rock, AR

## Abstract

**Background:**

Inflammatory bowel disease (IBD) incidence and prevalence have been increasing worldwide with higher disease severity observed in pediatrics. Limited data exists on the effectiveness of ustekinumab (UST) in children.

**Aims:**

To describe the effectiveness and safety of UST in pediatric patients with IBD.

**Methods:**

A retrospective, single-center cohort study was conducted between January 2017 and February 2022. The study included patients ≤16 years of age who were treated with off-label UST and followed up for a minimum of one year. Clinical remission was defined as a decrease in the score on the Pediatric Crohn's Disease (CD) and Pediatric Ulcerative Colitis (UC) Activity Indices to ≤10 at week 52.

**Results:**

A total of 13 patients who had failed anti-TNFα therapy were included, 8 (61.5%) with CD and 5 (38.5%) with UC. The median age of the patients was 13y (IQR: 11.5 to 14). UST treatment was initiated at a median of 3y (IQR: 2.3 to 7) after diagnosis. Ten patients (76.9%) achieved clinical remission. There were no statistically significant differences in baseline characteristics between patients who achieved and did not achieve clinical remission. Biochemical remission was achieved in 6 patients (46.2%). C-reactive protein levels decreased significantly in the remission group and insignificantly in the non-remission group. No significant changes were observed in erythrocyte sedimentation rate, albumin, or hemoglobin. BMI improved (Fig 1) and the need for corticosteroids significantly decreased after UST therapy in the remission group (P = 0.014). Endoscopy conducted post-treatment in 7 patients confirmed remission in 6 patients, with one patient showing clinical remission but not endoscopic remission. Adverse events included two instances of infection and once case of headache.

**Conclusions:**

UST was effective as a secondary biologic therapy for the induction and maintenance of remission in patients with anti-TNFα refractory IBD. At one year, 84% of patients remained on UST with no severe adverse reactions reported. Future prospective large-scale studies are necessary to assess the therapeutic positioning and optimal dosing regimens of UST in pediatric IBD.

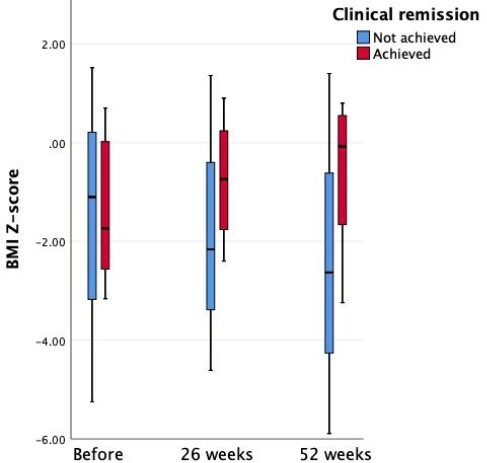

Figure 1. Change in BMI Z-score throughout the study (P value of interaction = 0.049).

**Funding Agencies:**

None

